# Evaluation of Topical Tocopherol Cream on Cutaneous Wound Healing in Streptozotocin-Induced Diabetic Rats

**DOI:** 10.1155/2012/491027

**Published:** 2012-10-14

**Authors:** Teoh Seong Lin, Azian Abd Latiff, Noor Aini Abd Hamid, Wan Zurinah bt Wan Ngah, Musalmah Mazlan

**Affiliations:** ^1^Department of Anatomy, Faculty of Medicine, Universiti Kebangsaan Malaysia, Jalan Raja Muda Abdul Aziz, 50300 Kuala Lumpur, Malaysia; ^2^Faculty of Medicine, Cyberjaya University College of Medical Sciences, 63000 Cyberjaya, Malaysia; ^3^Department of Biochemistry, Faculty of Medicine, Universiti Kebangsaan Malaysia, Jalan Raja Muda Abdul Aziz, 50300 Kuala Lumpur, Malaysia

## Abstract

Diabetes is a common cause of delayed wound healing. The aim of the study was to determine the effect of topical administration of tocopherol cream on the wound healing process in diabetic rats. The study was conducted using 18 male Sprague Dawley rats which were divided into three groups: (I) diabetic rats receiving control cream (*n* = 6), (II) diabetic rats receiving 0.06% tocopherol cream (*n* = 6), and (III) diabetic rats receiving 0.29% tocopherol cream (*n* = 6). Four cutaneous wounds were created at the dorsal region of the rats. Wound healing was assessed by total protein content, rate of wound closure estimation, and histological studies on the tenth day after wounding. Tocopherol treatment enhanced the wound healing process by increasing rate of wound closure and total protein content significantly (*P* < 0.05) compared to the control group. Histological observation also showed better organized epithelium and more collagen fibers in the tocopherol treated groups. Application of tocopherol cream enhances wound healing process in diabetic condition which is known to cause delay in wound healing.

## 1. Introduction

Wound can be defined as a disruption of the normal cellular, anatomical, and functional continuity of a structure. Thus, wound healing is a complex process which aims to restore the structural and functional integrity of the wounded tissue [1]. Wound healing can be divided into 3 stages, inflammation, proliferation, remodeling and maturation phases which involved the interaction of various cells, cytokines, and growth factors [2]. In some pathological disorders like diabetes mellitus, renal failure, malnutrition, wound healing is greatly impaired [3]. In diabetic patients, the prevalence of diabetic foot ulcers are 4–10%, and the treatment of foot ulcers are expensive and extensive [4]. Previous research study has shown that free radical inhibits the wound healing process [5]. Thus, the wound healing process can be accelerated by using antioxidants. 

Recently, research has focused on the use of natural antioxidants like herbal extracts and vitamins on wound healing. The beneficial effects of vitamins on wound healing have mainly been studied using animal models. Only vitamin C has been shown to accelerate healing in human subjects [6]. Oral and topical application of vitamin A has been shown to enhance healing in diabetic, immunocompromised, and malignant tumor animal models [7–10]. The positive effect of vitamin E oral administration on wound healing has also been well documented. Previously, we had reported benefit of oral administration of palm-vitamin E on wound healing in aging and diabetic rat models [11, 12]. Raxofelast, a hydrophilic vitamin-E-like compound injected intraperitoneally has shown its promising wound healing properties in an incisive wound model of diabetic rats [13]. However, considering the poor effusion and microcirculation insufficiency in diabetic patients, topical application of vitamin E might be more effective in accelerating wound healing compared to oral administration. Hence, in this study, we aimed to evaluate the effect of tocopherol topical application in the form of cream on wound healing of streptozotocin-induced diabetic rats.

## 2. Methods

### 2.1. Animals

Healthy male Sprague-Dawley rats (weighing 250–300 g) bred in Laboratory Animal Resource Unit, Universiti Kebangsaan Malaysia were used throughout the experimental period. They were housed under controlled environmental conditions with free access to rat pellets and clean water, caged individually. Prior ethical approval was obtained from the Universiti Kebangsaan Malaysia Animal Ethics Committees (UKMAEC). 

### 2.2. Diabetes Induction

Streptozotocin (STZ, Sigma, Germany) was dissolved in normal saline. Following this, 45 mg/kg dose of STZ was injected to the overnight fasted rats via lateral tail vein under mild diethyl ether anesthesia [2]. Three days later, blood samples were drawn from the tail of these rats to determine fasting blood glucose level using glucometer (Advantage, Germany). The rats with fasting blood glucose levels more than 8 mmol/L were labeled diabetic and were included for the experiment. 

### 2.3. Experimental Groups

The diabetic rats were randomly divided into 3 groups with 6 rats in each group. Group I considered as diabetic control group receiving vehicle cream. Whereas group II and III served as diabetic treatment group, treated with 0.06% and 0.29% tocopherol cream (Golden Hope Bioorganics, Malaysia), respectively. 

### 2.4. Rat Excision Wound Model and Treatment

The fur on the back of the anesthetized rats was shaved with electrical shaver and cleaned with alcohol swab. Four full thickness excision wounds were made on the dorsum of each rat with sterile 6 mm punch biopsy needles (Stiefel, Ireland) [12]. The excision included the epidermis, dermis, and panniculus carnosus. The wounds were left undressed and were treated with 0.1 g cream according to their respective groups topically once daily for a period of 10 days.

### 2.5. Rate of Wound Closure Determination

The wound with a centimeter scale was photographed after wounding day 0, day 6, and day 10. The photographs were used for the measurement of the wound areas using image analyzer software [2]. The rate of wound closure, that is, percentage of wound reduction from the original wound was calculated using the following formula [14]:

(1) Rate  of  wound  closure=wound  area  day  0−wound  area  day  (n=1,6,10)×100%  wound  area  day  0.

### 2.6. Termination of Experiment

After 10 days of daily treatment of topical cream (20 days after STZ), the rats were euthanized by cervical dislocation under anesthesia. A pair of wound was excised using the same-sized punch biopsy needle and stored at −70°C for Bradford assay. The other pair was excised, along with a 5 mm margin of the surrounding unwounded skin preserved in 10% formalin for light microscopy. 

### 2.7. Bradford Assay

Bradford method was used for quantitation of total protein content in the wound [15]. The wound tissue was homogenized in 1.15% potassium chloride at a ratio of 1 : 5 (wt/vol). Five mL of protein reagent (0.01% Coomassie Brilliant Blue G250, 4.7% ethanol, 8.5% phosphoric acid) was added and mixed into 0.1 mL of homogenate. Absorbance was measured at 595 nm against a blank protein reagent prepared by mixing 0.1 mL normal saline and 5 mL protein reagent. A standard curve was prepared using 0, 20, 40, 60, 80, and 100 *μ*g/mL of BSA in normal saline treated similarly.

### 2.8. Histological Observation

The formalin-fixed tissues were routinely processed by standard procedures and serial sections of 5 *μ*m were cut and stained with hematoxylin and eosin (H&E) and Verhoeff's van Gieson (VVG). The slides were examined and photomicrographs were obtained using digital camera (Pixelink, Canada) attached to a light microscope (Leica, Germany). 

### 2.9. Statistical Analysis

All results were expressed as mean ± SEM. The means of the group was compared using analysis of variance (ANOVA) followed by Scheffe's test. Data analysis was performed using statistical package programme SPSS 11.5. A *P* value <0.05 was considered as statistically significant.

## 3. Results

### 3.1. Diabetic Induction

**Figure 1:**
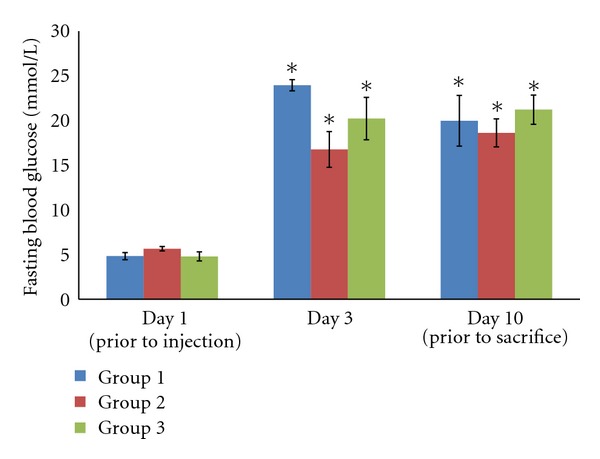
Fasting blood glucose level of the experimental rats. The rats experience elevated blood glucose level three days after the injection of STZ, and remain high throughout the experimental period (*P < 0.01).

Following injection of STZ intravenously, the rats showed 3-4-fold increase of fasting blood glucose level compared to their normal level before injection (Figure 1, *P* < 0.01). The fasting blood glucose level remained elevated throughout the experimental period. Topical treatment given to the rats wound did not exhibit any effect on the fasting blood glucose level (*P* > 0.05). 

### 3.2. Rate of Wound Closure

**Figure 2:**
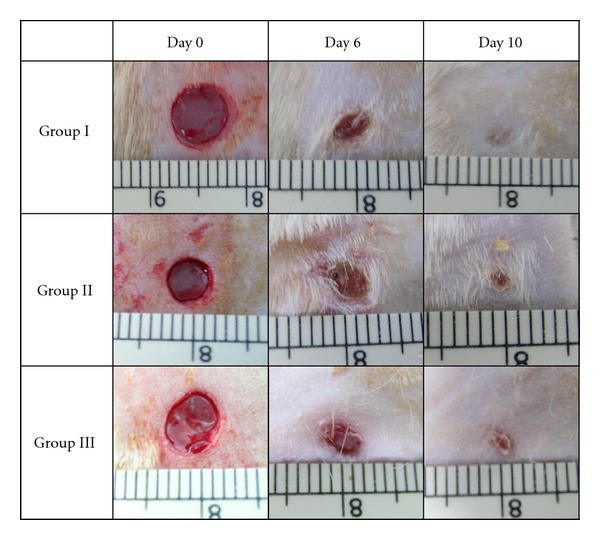
Photograph taken on the excision wounds made on the experimental rats. All groups showed complete wound closure on day 10.

**Figure 3:**
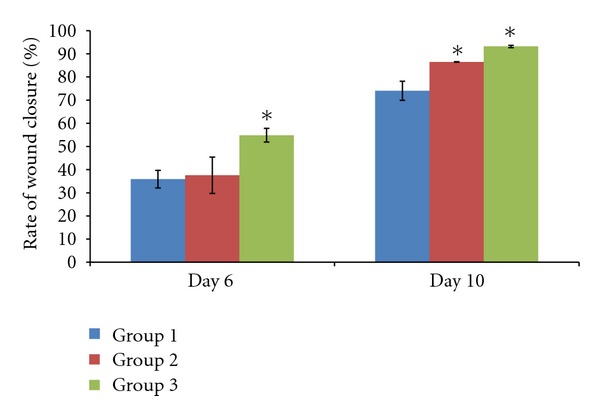
Effect of tocopherol on rate of wound closure. Rate of wound closure was measured as percentage of reduction of original wound size. Topical tocopherol treatment given to the diabetic rats increased the rate of wound closure (*P < 0.05). Treatment with 0.29% tocopherol cream showed higher rate of wound closure compared to the 0.06% tocopherol cream.

Figure 2 showed the contraction rate of the wounds on different days of the experimental rats. Rate of wound closure was calculated as the percentage of wound reduction from the original wound (Figure 3). The rate of wound closure was significantly increased in both the diabetic rat groups receiving treatment (*P* < 0.05). Diabetic rats receiving treatment of 0.29% tocopherol cream have a higher rate of wound closure compared to the 0.06% tocopherol cream. 

### 3.3. Bradford's Assay

**Figure 4:**
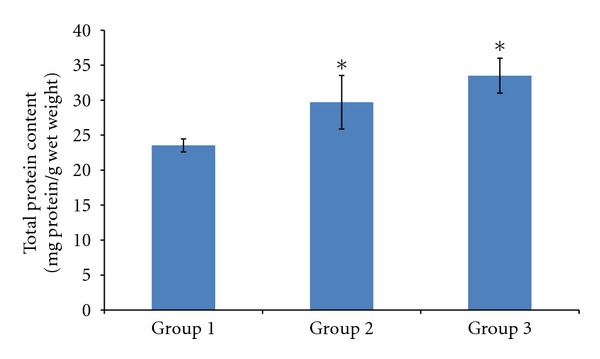
Effect of tocopherol on total protein content. Total protein content was measured according to Bradford's assay. There was significant increase in the total protein content in the wound tissues harvested when given treatment with tocopherol cream (*P < 0.05). Treatment with 0.29% tocopherol cream showed the highest total protein content amongst other groups.

Total protein content of the wounds (Figure 4) harvested from experimental rats on day 10 was estimated using Bradford's assay. Treatment with tocopherol cream increased the total protein content of the wound significantly (*P* < 0.05). 

### 3.4. Histological Findings

**Figure 5:**
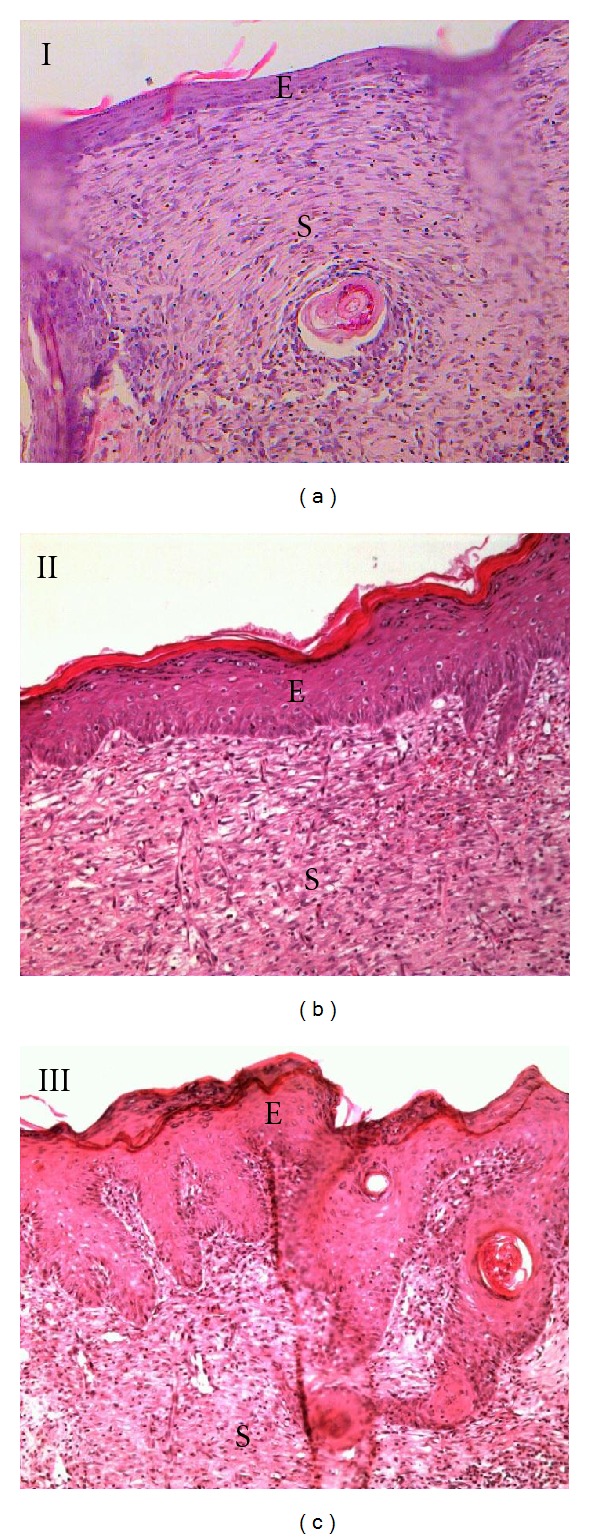
H&E stained section on the 10th day wound (10X). Complete epithelialization of the wound area was observed in all groups. Newly formed epithelium in group I appeared thin. There were interdigitation between epithelium and dermis of the skin in group III suggesting stronger integrity of the skin. E: epidermis and S: scar/granulation tissue.

**Figure 6:**
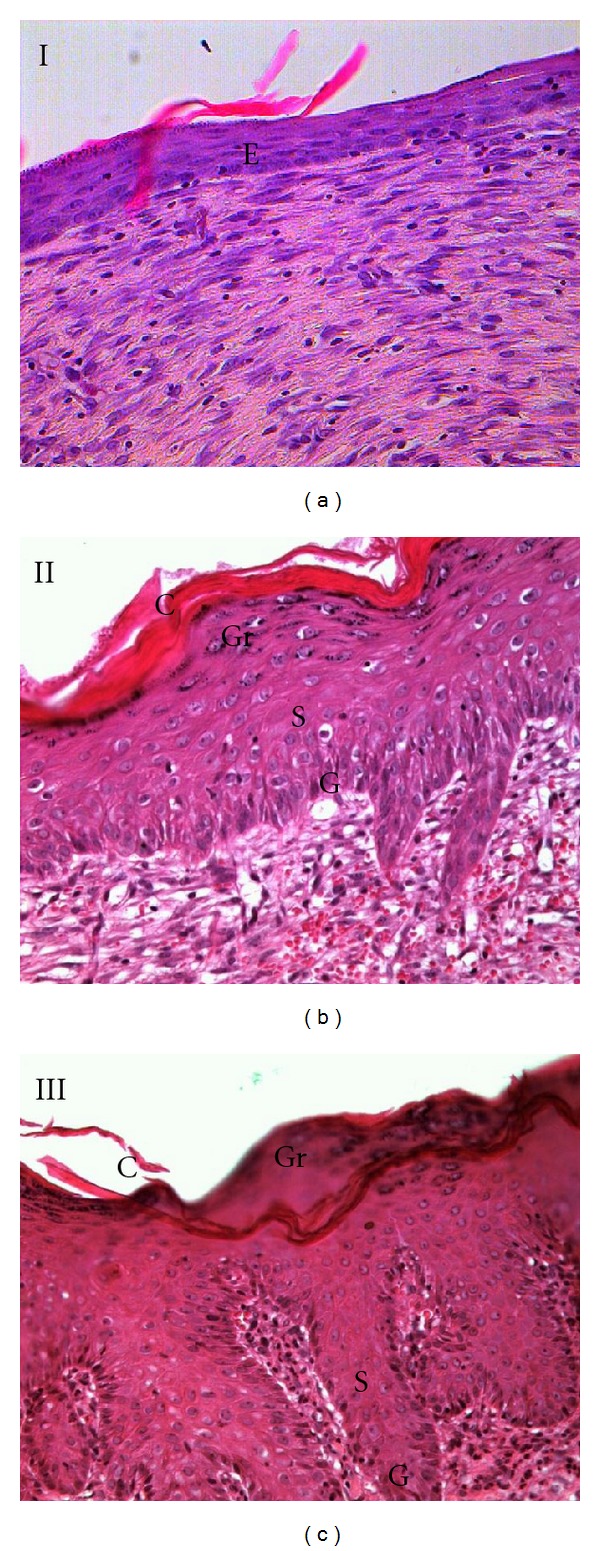
H&E stained section on the 10th day wound (20X). Evaluation on the newly formed epithelium revealed that group I does not contain all the strata of an epithelium. Whereas group II and III showed the presence of strata germinativum (G), spinosum (S), granulosum (Gr), and corneum (C). E: epidermis.

**Figure 7:**
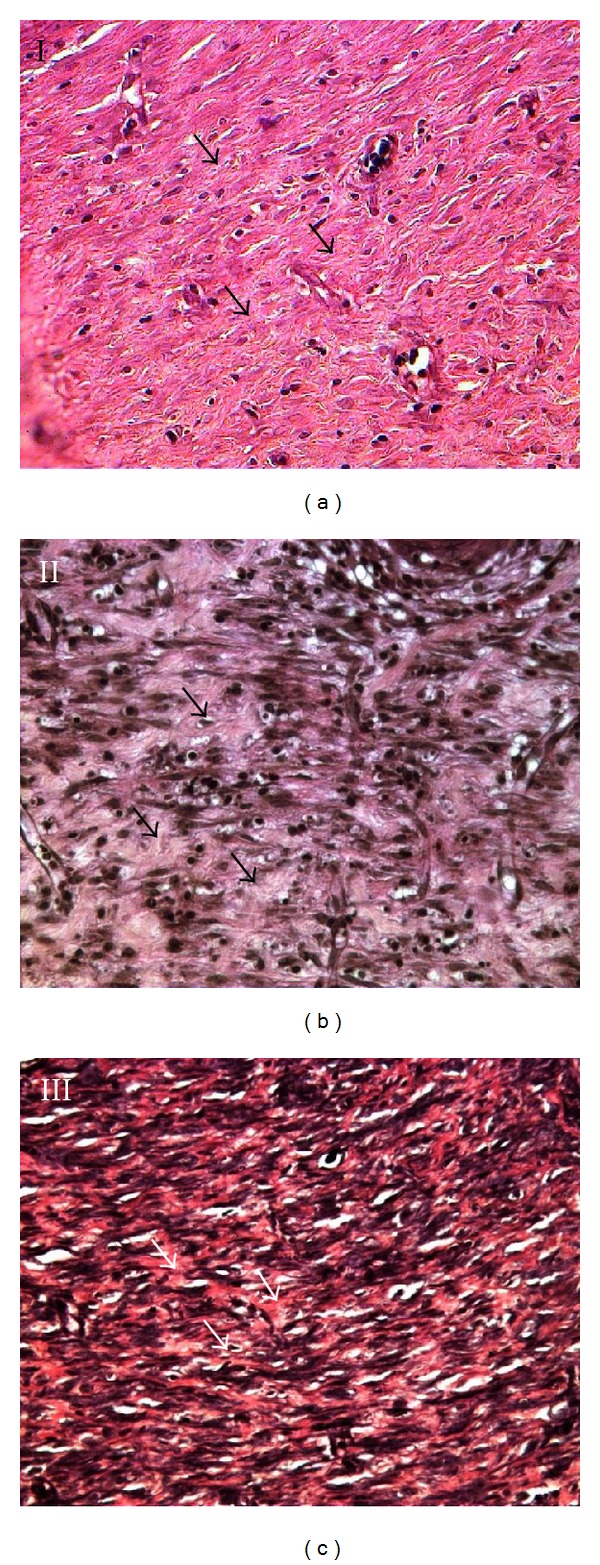
VVG stained section on the 10th day wound (40X). The collagen fibres (arrows) found in the scar tissue deep to the epithelium in group I appeared thin and scanty. Treatment with tocopherol cream increased the abundance of collagen fibres found in the scar tissue.

H&E staining was performed on the wounds harvested on day 10 (Figures 5 and 6). All groups of experimental rats showed complete epithelialization of the wound area. The new epithelial layer formed in group I appeared thin and did not show all four strata structure. Tocopherol cream treated group showed a well-formed epithelium. Treatment with 0.29% tocopherol cream increased the interdigitation between the epithelium and dermis layers. VVG staining (Figure 7) showed the abundant collagen fibers formed in both the treated group compared to the scanty deposition of fibres in the control group. 

## 4. Discussion

Tocopherol and tocotrienol which are structurally similar are subfamily of vitamin E [12]. *α*-Tocopherol is known as the most abundant and active form of vitamin E in humans [16, 17]. Previous studies have demonstrated the beneficial effects of vitamin E in wound healing process when given orally [11, 12]. The present study shows that topical application of tocopherol cream also enhances wound healing in diabetic rats. 

In this present study, STZ was used to induce diabetes in experimental rats intravenously. Three days after the injection of STZ, all the rats showed increase of fasting blood glucose level, reduced body weight, polydipsia, and polyuria. The fasting blood glucose remained constantly elevated during the entire experimental period. These sign and symptoms were shown in a previous study as well [18]. The topical application of tocopherol cream in group II and III exhibited no effect on the fasting blood glucose. 

Rate of wound closure is a useful measurement to assess the progress of wound healing. It had been shown that there is a decrease in rate of wound contraction in diabetic rats compared to the normal rats [2]. In the present study, application of both 0.06% and 0.29% tocopherol cream was able to increase the rate of wound contraction. This is in accordance to a previous study which showed increased rate of wound contraction following oral supplementation of tocopherol [12]. 

The total protein content is an indicator for the protein level and cellular proliferation of the wound tissue [2]. In this study, tocopherol cream was able to increase the total protein content in the diabetic rats, which is in accordance to a previous study [12]. This could indicate that tocopherol enhances protein synthesis, cellular proliferation, and migration in the wound tissue. Interestingly, a previous study showed that physiologically relevant concentrations of *α*-tocopherol inhibits cell proliferation in vascular smooth muscle cells while proliferation of fibroblast was not inhibited [19]. 

Histological observation revealed complete epithelialization of all diabetic rats, with or without tocopherol cream treatment after 10 days of wounding. However, untreated diabetic rats showed immature and thin epithelial layer. Tocopherol treated group showed that the newly formed epithelium contains the strata germinativum, spinosum, granulosum, and corneum. In group III, there was presence of interdigitation between the epithelium and dermis. These interdigitations were important as it offered resistance to separation of the epidermal layer due to shreding. It was also shown that with tocopherol treatment, collagen fibers were more numerous in the scar tissue which was also reflected in the total protein content. 

Diabetes mellitus, a common endocrine disease, is one of the leading causes of impaired healing. Delayed wound healing in diabetes is multifactorial which includes hyperglycaemia, infections, suppressed immunity, local ischemia, and oxidative stress [20]. In diabetic condition, hyperglycaemia decreases cell proliferation and collagen deposition. The chronic inflammation which occurs at the wound site causes local ischemia, generation of reactive oxygen species (ROS), vascular stasis in microcirculation, decreased chemotaxis and phagocytosis, reduced level of growth factors, inhibition of fibroblast proliferation, and decreased deposition of extracellular matrix molecules which then leads to delay of wound healing in diabetic patients [20, 21]. Previous study has shown that tocopherol stimulates the production of cyclic adenosine monophosphate (cAMP) which often associated with immunomodulatory effects by modulating the inflammatory responses of a variety of immune cell types including macrophages [22]. Tocopherol also has been shown to possess anti-inflammatory effect by attenuation of pro-inflammatory cytokine and chemokine production [22]. Thus, application of tocopherol is beneficial to wound healing in diabetic rats which might be due to its antioxidant and anti-inflammatory property. 

In conclusion, the administration of topical tocopherol cream has positive effect on wound healing process in diabetic animal model. In the present study, the higher dose of 0.29% tocopherol cream showed better wound healing compared to the 0.06% tocopherol cream.
